# Analysis of Midgut Bacterial Communities in Larvae and Adult Mosquitoes of *Aedes aegypti* Invaded by Three Different Microorganisms

**DOI:** 10.3390/microorganisms13020248

**Published:** 2025-01-23

**Authors:** Yanan Yin, Yanhui Liu, Jieli Fan, Lingling Yu, Meng Nie, Zhiqi Zhang, Qian Han, Chenghong Liao

**Affiliations:** 1 Laboratory of Tropical Veterinary Medicine and Vector Biology, School of Life and Health Sciences, Hainan Province Key Laboratory of One Health, Collaborative Innovation Center of One Health, Hainan University, Haikou 570228, China; yinyanan54@163.com (Y.Y.); yanhuiliu11@hotmail.com (Y.L.); fanjieli0722@163.com (J.F.); gzzljszyyx@163.com (L.Y.); 23220860010011@hainanu.edu.cn (M.N.); zhangzhiqi202311@163.com (Z.Z.); qianhan@hainanu.edu.cn (Q.H.); 2 Hainan International One Health Institute, Hainan University, Haikou 570228, China

**Keywords:** mosquito microbiota, *Escherichia coli*, *Staphylococcus aureus*, *Beauveria bassiana*

## Abstract

The midgut microbiota of *Aedes aegypti* is crucial for the mosquito’s development, nutrition, and immunity. However, its communities are also distinctively influenced by the colonization of different microorganisms, influencing its susceptibility to pathogens and transmission capacity. In this study, we investigated the effects of infections with *Escherichia coli*, *Staphylococcus aureus*, and *Beauveria bassiana* on the midgut microbial composition of *Ae. aegypti*. These microorganisms were inoculated into the midguts of third-instar larvae using a soaking method. Midgut samples were then analyzed through high-throughput 16S rDNA sequencing to assess bacterial load and microbiota composition of fourth-instar larvae and female adult mosquitoes. The results reveal that *E. coli*-colonized fourth-instar larvae (CO_4W) exhibited 20 unique genera, whereas the *S. aureus*-colonized group (S_4W) had operational taxonomic units assigned to 194 bacterial taxa, including a notable decrease in *Elizabethkingia*. In addition, *B. bassiana* infection led to a significant reduction of *Elizabethkingia meningoseptica* in larvae, decreasing from 42.9% in the control group (CK_4W) to 0.9% in the *B. bassiana*-infected group (B_4W). Distinct microbial profiles were also compared between adult mosquitoes and fourth-instar larvae. Significant abundance changes were found in Firmicutes, Bacteroidota, and Proteobacteria among different groups. Metabolic pathway predictions using PICRUSt suggested that microorganism invasion enriched the pathways involved in carbohydrate metabolism and amino acid metabolism. This enrichment suggests that the microbiota may undergo specific adaptive responses to pathogen presence. Overall, our results provide new insights into the relationship between the invasion of microorganisms and midgut bacterial communities in mosquitoes.

## 1. Introduction

The microbiota of mosquitoes plays a crucial role in their development and survival, significantly influencing host phenotypic traits and susceptibility to pathogens [1,2,3]. Previous studies have not only revealed subtle differences in midgut microbiota at the phylum and family levels between wild and laboratory-reared mosquitoes but have also highlighted the role of gut bacteria in influencing the mosquito infection, tolerance, and transmission capacity of pathogenic arthropod-borne viruses [4,5,6,7,8,9].

One of the most studied bacteria in this context is *Escherichia coli* (*E. coli*), a common gut flora [10,11]. *E. coli* has been shown to impact mosquito survival and vector competence, influencing the microbial balance in the midgut and potentially affecting pathogen infection dynamics [12]. Research has found that exposure to *E. coli* significantly reduces the prevalence and intensity of *Plasmodium falciparum* colonization in mosquitoes, potentially leading to a disruption of gut homeostasis [13]. The invasion of *E. coli* often results in an increased abundance of Enterobacteriaceae, the bacterial family to which *E. coli* belongs. Conversely, studies have found that certain microorganisms, such as Enterobacteriaceae and *Serratia odorifera*, can facilitate or enhance viral infections in mosquitoes [14]. In addition, *E. coli* colonization can trigger immune responses in mosquitoes, leading to the production of antimicrobial peptides and other immune effectors [15,16]. Not only can this immune activation influence the bacterial load in the gut, but natural genetic variation in immune-related genes can also shape, with high specificity, the bacterial community structure in the mosquito gut [17,18]. Similarly, studies have shown that *S. aureus* colonization leads to changes in the immune response and a noticeable reduction in the diversity of gut microbial communities. These changes can modulate the ability of mosquitoes to defend against and transmit pathogens [15,16].

Entomopathogenic fungi, such as *Beauveria bassiana* (*B. bassiana*), have gained attention as promising next-generation agents for mosquito control [19,20]. Additionally, fungal infections have been shown to increase midgut microbiota diversity while altering the relative abundance of specific bacterial groups. Notably, *B. bassiana* has been associated with increased mosquito mortality, a process believed to result from its interaction with the midgut microbiota [21,22]. Specifically, *B. bassiana* infection has been found to be associated with a decrease in certain bacterial genera, such as *Pseudomonas* and *Enterobacter* [23,24], highlighting how fungal infections can disrupt the microbial balance in the mosquito gut and potentially influence mosquito health and pathogen resistance [21].

This variability underscores the need for more targeted, case-specific analyses to better understand the differences in microbiota composition and function. Recent studies have demonstrated that the microbiota plays a crucial role in shaping mosquito pathogen transmission capabilities and host phenotypes [15,16,25,26]. Given that mosquito survival is influenced by *E. coli*, *S. aureus*, and *B. bassiana*, we sought to explore whether the effects of these pathogens on mosquito survival are linked to changes in the gut microbiota. Understanding these intricate host–pathogen–microbiota interactions is critical for the development of effective biocontrol strategies that target specific microbial communities.

## 2. Materials and Methods

### 2.1. Experimental Preparation and Mosquito Rearing

*Ae. aegypti* eggs were reared to adulthood under standard laboratory conditions—maintained at a temperature of 28 ± 2 °C, a relative humidity of 75 ± 5%, and with a 12:12 h light–dark photoperiod. The eggs were hatched in dechlorinated water, and the resulting larvae were fed with sterilized mouse feed. To ensure a clean environment, the dechlorinated water was regularly replaced throughout the larval development phase. Upon reaching the pupal stage, the larvae were transferred to paper cups, and the pupae were isolated to prevent overcrowding. Upon maturation into adult mosquitoes, they were sustained on an 8% sterilized sucrose solution. Sterile paper cups were filled with double-distilled water, each containing approximately 100 third-instar larvae, to be used for subsequent microbial inoculation.

Four experimental groups were established as follows: Group A (CK_4W), consisting of non-colonized larvae; Group B (CO_4W), comprising larvae colonized with *E. coli*; Group C (S_4W), including larvae colonized with *S. aureus*; and Group D (B_4W), consisting of larvae infected with *B. bassiana*. The bacterial suspension for each group was refreshed daily until the larvae developed into adults. The adult mosquito groups were designated as CK_CW, CO_CW, S_CW and B_CW, (“CK,” the control group; “CO,” the *E. coli* colonization group; “S”, the *S. aureus* colonization group; “B”, the *B. bassiana* infection group; “CW”, adult mosquitoes).

To minimize contamination, the surfaces of both fourth-instar larvae and adult mosquitoes were sterilized using 75% ethanol for 1 min, followed by three 30 s rinses in sterile water. Midgut dissections and collections were conducted under sterile conditions. For each sample, a total of 100 larval midguts were pooled and rapidly frozen in liquid nitrogen for subsequent analysis. Each experimental group (CK_4W, CO_4W, S_4W, B_4W, CK_CW, CO_CW, S_CW, B_CW) included three biological replicates, with each replicate consisting of midguts pooled from 100 larvae or adults. The sample size was determined based on previous microbiome studies in mosquitoes, which have successfully identified significant differences with three biological replicates per group [27].

### 2.2. Preparation of Microorganisms

Aliquots of *E. coli* (DH5α, Shanghai Weidi Biotechnology Co., Ltd., Shanghai, China) were inoculated in Luria–Bertani liquid medium, while *S. aureus* (BNCC330041, BeNa Culture Collection) was cultured in tryptic soy broth (TSB), with both incubated overnight for 12–16 h at 37 °C in a shaking incubator set to 180 rpm. The microbial suspensions were centrifuged at 8000× *g* for 30 min at 4 °C, and the supernatant was discarded. The resulting bacterial pellets were resuspended in sterile water and adjusted to a concentration of 10 mg/mL (*w*/*v*) for each strain [28]. *B. bassiana* (BNCC192026, BeNa Culture Collection) strains were cultured on potato dextrose agar (PDA) medium, composed of 1.0 L potato broth, 20.0 g glucose, 3.0 g KH_2_PO_4_, 1.5 g MgSO_4_·7H_2_O, trace amounts of Vitamin B1, and 20.0 g agar, at 28 °C for three to seven days. Activated colonies were then transferred to a liquid culture medium containing 4% D-glucose and 1% peptone and were cultured at 28 °C and 180 rpm for three days. The fungal solution was filtered, and the spore suspension was diluted to 1 × 10^5^ spores/mL for subsequent use.

Groups of 100 third-instar larvae were manually counted and transferred to sterile plastic cups, with 15 cups per group. Under laboratory conditions, the control group was provided with 150 mL of sterile water, while the three experimental groups were each given 150 mL of *E. coli* suspension, *S. aureus* suspension, or spore suspension. Fresh sterile water, bacterial suspensions, and spore suspensions were replaced daily for the larvae until they had developed into adults. The same larval density was used in all experiments [28].

### 2.3. PCR Amplification and Sequencing

Microbial DNA was extracted using HiPure soil DNA kits (Magen, Guangzhou, China) following the manufacturer’s instructions. The full length 16S rDNA was amplified by PCR using the following conditions: initial denaturation at 95 °C for 2 min, followed by 35 cycles of 95 °C for 30 s, 60 °C for 45 s, and 72 °C for 90 s, with a final extension at 72 °C for 10 min. The primers used were 341F (CCTACGGGNGGCWGCAG) and 806R (GGACTACHVGGGTATCTAAT) [29]. The PCR reaction was conducted in a 50 μL volume with TransGen High-Fidelity PCR SuperMix (TransGen Biotech, Beijing, China), 0.2 μM forward and reverse primers, and 5 ng template DNA. The amplicons were extracted from 2% agarose gels and purified using the AxyPrep DNA gel extraction kit (Axygen Biosciences, Union City, CA, USA) according to the manufacturer’s instructions. The purified amplicons were quantified using an ABI StepOnePlus real-time PCR system (Life Technologies, Foster City, CA, USA). Finally, the purified amplicons were pooled in equimolar amounts and were paired-end sequenced (PE250) on an Illumina platform following the standard protocols (Guangzhou Genedenovo Biotechnology Co., Ltd., Guangzhou, China).

### 2.4. 16S-rDNA Sequencing Data Analysis

Raw sequencing reads were processed using FASTP [30] (version 0.18.0) to obtain high-quality clean reads by filtering out reads with more than 10% unknown nucleotides and those with less than 50% of their bases having a quality score (Q-value) greater than 20. Paired-end clean reads were then merged using FLASH [31] (version 1.2.11), with a minimum overlap of 10 base pairs and a mismatch error rate of 2%. Noisy sequences were filtered under specific conditions to ensure the generation of high-quality clean tags [32]. These clean tags were clustered into operational taxonomic units (OTUs) with ≥97% similarity using the UPARSE pipeline (version 9.2.64), and chimeric tags were removed using the UCHIME algorithm [33]. The most abundant tag sequence within each cluster was selected as the representative sequence.

Taxonomic classification of the representative OTU sequences was performed using the RDP classifier [34] (version 2.2) based on the SILVA database [35] (version 138.1), with a confidence threshold of 0.8. Microbial diversity and community composition were analyzed on the Gene Denovo Biotechnology online platform (www.Omicsmart.com, accessed on 18 January 2025). For taxonomic classification, BLAST [36] (version 2.6.0) was used to compare OTU sequences against the NCBI 16S ribosomal RNA database. Optimal thresholds for classifications were determined through an empirical statistical model with a receiver operating characteristic curve [37].

The species diversity in the groups is presented using a rank abundance curve. Differences among microbial communities were visualized using principal coordinate analysis (PCoA). Microbial biomarker features for each experimental group were identified using linear discriminant analysis effect size (LEfSe) software (version 1.0). Statistical differences between the pathogen-infected groups and control groups were evaluated using Welch’s *t*-test, with significance set at *p* < 0.05. All reads from this study are available at the NCBI under accession number PRJNA1188320.

## 3. Results

### 3.1. Structures of Midgut Bacterial Community in Larvae and Adults Colonized with E. coli

**Figure 1:**
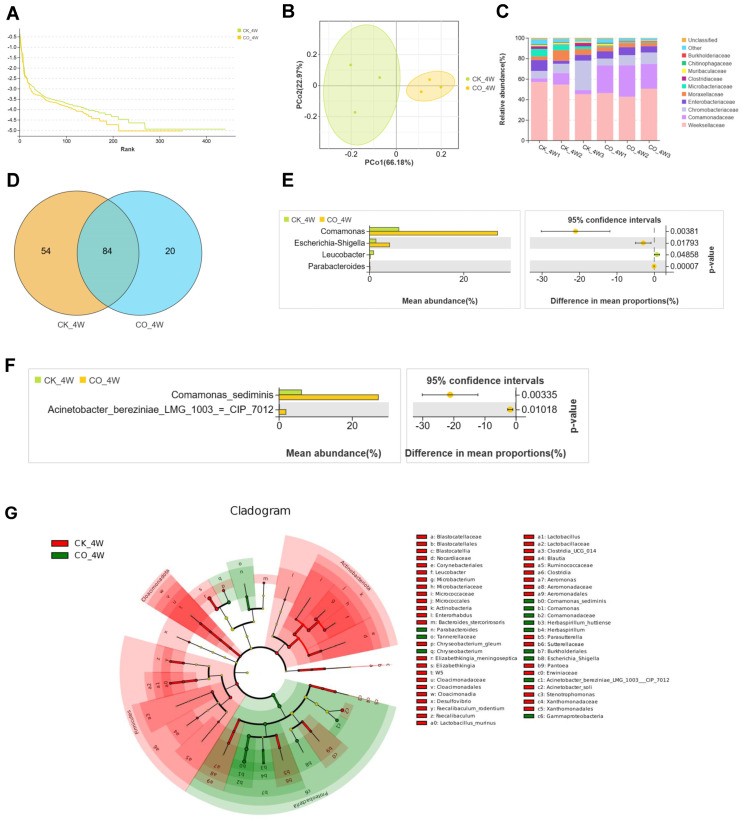
Composition and diversity of midgut bacterial microbiota in the fourth-instar larvae of *Aedes aegypti* colonized with *E. coli*: (**A**) Rank abundance curve; (**B**) PCoA of differences in the midgut microbiota between CK_4W and CO_4W; (**C**) community analysis of midgut bacteria, with the color-coded bar plot showing the average bacterial genus distribution in both groups; (**D**) comparative analysis of Venn plots between the two groups at the genus level; (**E**) relative abundance of genera with significant differences among samples between CK_4W and CO_4W (*p* < 0.05); (**F**) relative abundances of the species showing significant differences among samples between the two groups (*p* < 0.05); (**G**) LEFse analysis of intergroup microbial differences, with evolutionary branching diagram showing the classification levels from domain to species. Each small circle at different taxonomic levels represents a species, with the circle’s diameter proportional to its relative abundance.

The midgut microbiomes of fourth-instar larvae and adult mosquitoes, both colonized with *E. coli* and non-colonized controls, were compared with assess the bacterium’s effects on the microbiota. In the larval group, after quality filtering of the samples through high-throughput sequencing of universal bacterial and archaeal 16S rDNA genes, a total of 686,756 raw reads and 638,530 valid tags were obtained (Table 1). The rank abundance curve (Figure 1A) shows that the *E. coli*-colonized CO_4W group exhibited lower microbial species richness compared with the non-colonized CK_4W group, reflecting that bacterial colonization can impact the abundance of midgut microbiota. To further investigate the similarity in the composition of the midgut microbiota between the two groups, a principal coordinates analysis (PCoA) was conducted, demonstrating a clear separation in the microbiota structure between the CK_4W and CO_4W groups (Figure 1B). Subsequently, the mean relative abundances of the ten most abundant bacterial families shared between these two groups were calculated and plotted to identify taxa that were enriched or reduced in relative abundance in the CO_4W group compared with the CK_4W group. At the family level, both groups showed similar distributions, with Weeksellaceae being the most dominant family, accounting for 52.5% in CK_4W and 46.7% in CO_4W (Figure 1C). These families were further analyzed at the genus level to evaluate whether *E. coli* colonization influenced the composition and abundance of midgut microbiota.

The results reveal that, following *E. coli* colonization, the number of midgut microbiota-specific genera in the CO_4W group decreased significantly, with only 20 genera identified, including *Pedomicrobium*, *Mycoplasma*, *Novosphingobium*, *Buchnera*, *Dickeya*, *Solibacillus*, *Thermicanus*, *Streptomyces*, *Mucilaginibacter*, *Shewanella* and ten other genera. In contrast, the CK_4W group harbored 54 midgut microbiota-specific genera (Figure 1D). At the genus level, the abundance of *Comamonas*, *Escherichia-Shigella*, and *Parabacteroides* was significantly higher in the CO_4W group compared with the CK_4W group (27.2% vs. 6.2%, 4.3% vs. 1.4%, and 0.13% vs. 0.08%, respectively), with *Parabacteroides* present at low levels in both groups (Welch’s *t*-test, *p* < 0.05; Figure 1E).

**Figure 2:**
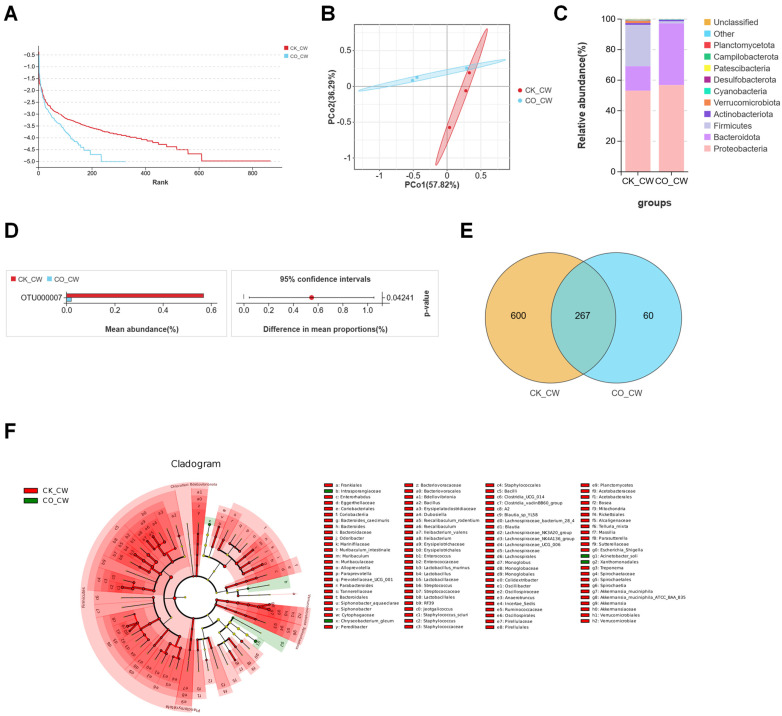
Composition and diversity of midgut bacterial microbiota in the adult mosquitoes of *Aedes aegypti* colonized with *E. coli*: (**A**) PCoA of differences in the midgut microbiota between CK_CW and CO_CW; (**B**) rank abundance curve; (**C**) community analysis of midgut bacteria, with the color-coded bar plot showing the average bacterial genus distribution in both groups; (**D**) relative abundances of OTUs with significant differences among samples between the two groups (*p* < 0.05); (**E**) comparative analysis of Venn plots between the two groups at the OTU level; (**F**) LEFse analysis of intergroup microbial differences, with evolutionary branching diagram showing the classification levels from domain to species. Each small circle at different taxonomic levels represents a species, with the circle’s diameter proportional to its relative abundance.

Additionally, several taxa, including *Comamonas sediminis* and *Acinetobacterbereziniae (LMG 1003* = *CIP 7012)* were found in low abundance in the CK_4W group but increased significantly in the CO_4W group (6.1% vs. 27.2%, 0.09% vs. 1.86%, respectively, as shown in Figure 1F). To explore the differences in bacterial taxa between the colonized and non-colonized larvae, this study conducted LEfSe analysis. This analysis identified 57 taxa with significant differences between the CK_4W and CO_4W groups, with 44 taxa enriched in the CK_4W group and 13 taxa enriched in the CO_4W group. At the genus level, the dominant taxa in the CK_4W group comprised 13 genera, including *Elizabethkingia* and *Microbacterium*. In the CO_4W group, the dominant taxa consisted of 5 genera, including *Comamonas* and *Chryseobacterium* (Figure 1G). In the adult mosquito group, 766,409 reads were obtained after quality filtering, resulting in the identification of 2552 OTUs (Table 1). In comparisons between adult mosquitoes colonized and non-colonized with *E. coli*, the rank abundance curves indicated that the *E. coli*-colonized CO_CW group exhibited lower microbial species richness compared with the non-colonized CK_CW group (Figure 2A), which is consistent with the findings observed in the larvae. To further investigate the relationship in the composition of the midgut microbiota between the two groups, a principal coordinates analysis (PCoA) was conducted, revealing no significant separation in the microbial community structure between the CK_CW and CO_CW groups (Figure 2B). At the phylum level, the microbiota of both the CK_CW group and the CO_CW group showed similar distributions, with the dominant phyla being Proteobacteria (53.2% and 56.8%) and Bacteroidota (16% and 40.4%) (Figure 2C).

Indicator species analysis showed that, among the non-colonized individuals, there was a significant difference in the abundance of OTU000007 (order: Pseudomonadales; family: Moraxellaceae; genus: *Acinetobacter*) between the two groups (Figure 2D). Additionally, 267 types of OTUs were shared between the colonized and non-colonized groups. However, more unique OTUs were observed in the CK_CW group than in the CO_CW group (Figure 2E). This finding suggests that *E. coli* colonization leads to a reduction in the diversity of the midgut microbiota, with fewer distinct OTUs in the colonized group. Furthermore, in the evolutionary branching analysis at the genus level, the CK_CW group exhibited 35 dominant genera, including *Enterococcus*, *Lactobacillus*, and *Staphylococcus*. In contrast, the CO_CW group did not display any dominant genera at the genus level (Figure 2F).

**Figure 3:**
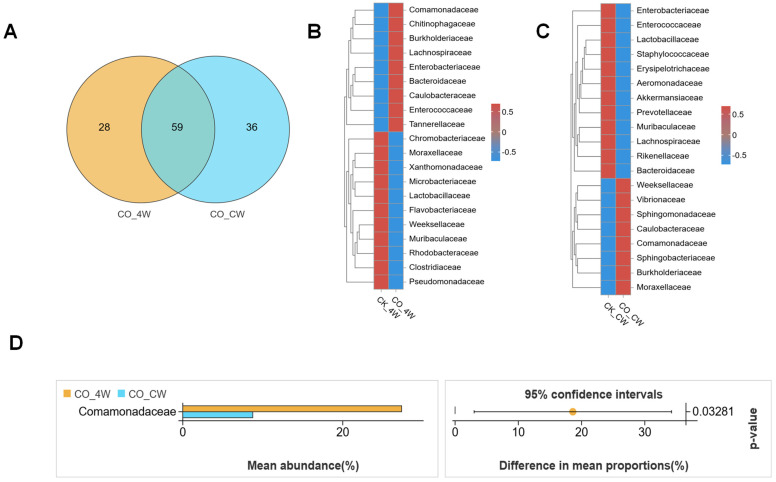
Compositional analysis of midgut bacterial communities in *Aedes aegypti* fourth-instar larvae and adults colonized with *E. coli*: (**A**) comparative analysis of VENN plots between the CO_4W and CO_CW at the family level; (**B**,**C**) heatmaps displaying the relative abundances of the two groups at the family level; (**D**) relative abundances of the bacterial families with significant differences among samples from the two groups (*p* < 0.05).

To compare the midgut microbiota between the fourth-instar larvae and adult *Ae. aegypti*, both with and without *E. coli* colonization, we performed an indicator species analysis at the family level. This analysis identified 59 bacterial families shared between the two life stages, including Weeksellaceae, Enterobacteriaceae, and Moraxellaceae. The larval group harbored 28 unique families, such as Rubritaleaceae, Arcobacteraceae and Mycoplasmataceae, while the adult group exhibited 36 distinct families, including Aeromonadaceae, Corynebacteriaceae and Devosiaceae (Figure 3A). The species heatmap at the family level showed that, after *E. coli* colonization, the midgut microbiota of both larvae and adult mosquitoes exhibited an increase in the abundance of Comamonadaceae, Caulobacteraceae, and Burkholderiaceae, while Lactobacillaceae and Muribaculaceae abundance decreased compared with the control group (Figure 3B,C). Notably, the relative abundance of Comamonadaceae showed a statistically significant increase (Welch’s *t*-test; *p* = 0.03281; Figure 3D). Within the mosquito midgut, bacteria from the Comamonadaceae family are hypothesized to play pivotal roles in the digestive processes, nutrient cycling, and the host’s immune defense against invading pathogens. As the colonization progresses, fluctuations in the population dynamics of Comamonadaceae may play a key role in supporting the mosquito’s immunological defenses and overall growth. These changes in abundance may reflect the adaptive strategies of this family within the mosquito’s microbiota, ultimately contributing to the host’s physiological homeostasis [38].

### 3.2. Structures of Midgut Bacterial Community in Larvae and Adults Colonized with S. aureus

**Figure 4:**
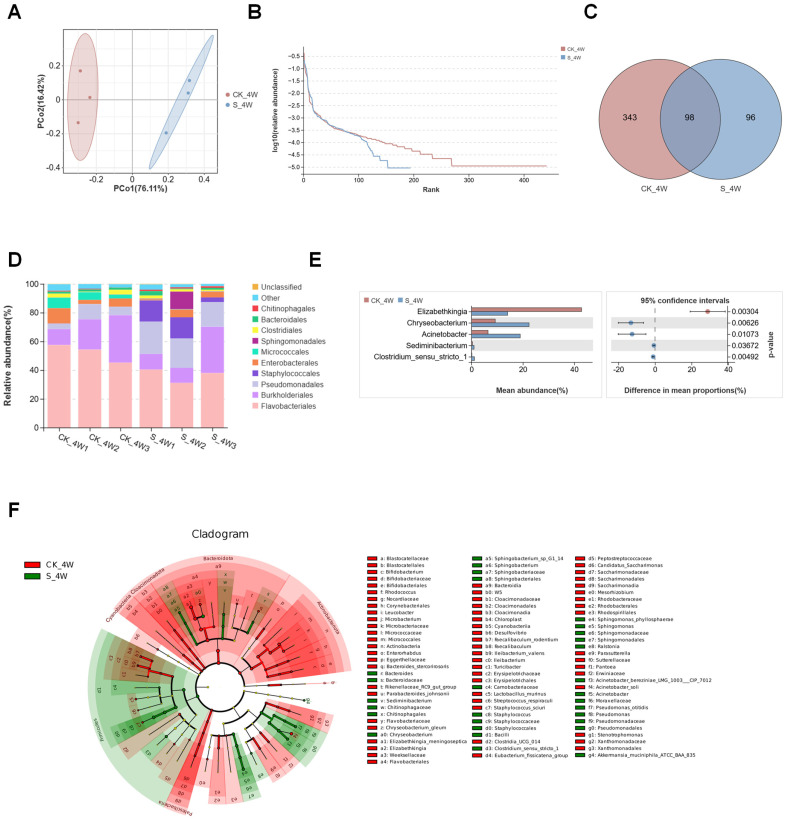
Composition and diversity of midgut bacterial microbiota in the fourth-instar larvae of *Aedes aegypti* colonized with *S. aureus*: (**A**) PCoA illustrating differences in the midgut microbiota between the CK_4W and S_4W groups; (**B**) rank abundance curve; (**C**) comparative analysis of Venn plots between the two groups at the OTU level; (**D**) community analysis of midgut bacteria., with the color-coded bar plot showing the average distribution of bacterial orders in the two groups; (**E**) relative abundances of the bacterial families with significant differences among samples between the two groups (*p* < 0.05); (**F**) LEFse analysis of intergroup microbial differences, with the evolutionary branching diagram showing the classification levels from domain to species. Each small circle at different taxonomic levels represents a species, with the circle’s diameter proportional to its relative abundance.

**Figure 5:**
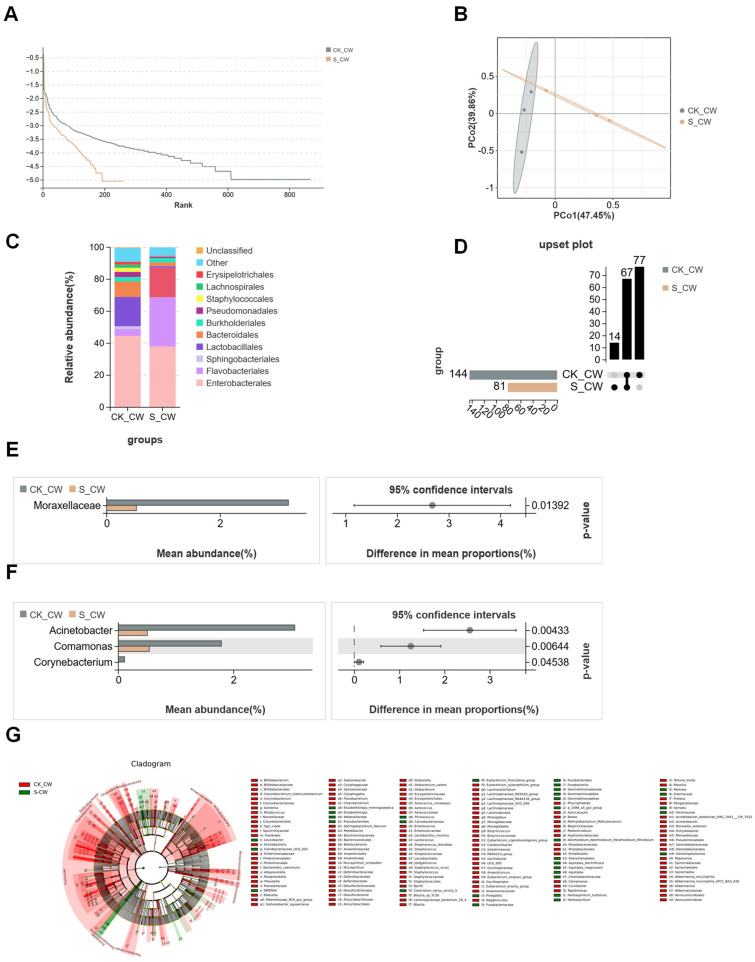
Composition and diversity of midgut bacterial microbiota in the adult mosquitoes of *Aedes aegypti* colonized with *S. aureus*: (**A**) Rank abundance curve; (**B**) PCoA illustrating differences in the midgut microbiota between the CK_CW and S_CW groups; (**C**) community analysis of midgut bacteria between the CK_CW and the S_CW, with the color-coded bar plot showing the average bacterial order distribution for both groups; (**D**) comparative analysis of family level Upset plots between the two groups; (**E**) relative abundances of the bacterial families with significant differences between the two groups (*p* < 0.05); (**F**) relative abundances of the bacterial genus with significant differences between the two groups (*p* < 0.05); (**G**) LEFse analysis of intergroup microbial differences, evolutionary branching diagram with circles radiating from the center representing classification levels from domain to species. Each small circle at different classification levels represents a species level, with the diameter of the circle proportional to its relative abundance.

To further explore the impact of bacterial colonization on the midgut microbiota, this study analyzed the midgut microbiota of fourth-instar *Ae. aegypti* larvae, comparing those colonized with *S. aureus* with non-colonized controls. After quality control of the resulting sequencing reads, 625,466 effective tags were identified (Table 1). PCoA confirmed that *S. aureus* colonization influenced the diversity of the bacterial communities, with the midgut microbiota clustered according to the presence or absence of *S. aureus* colonization, indicating distinct community structures between the two groups (Figure 4A). The rank abundance curves further demonstrated that *S. aureus* colonization had led to a reduction in the species richness of the midgut microbiota (Figure 4B). Overall, OTUs from the *S. aureus*-colonized larvae (S_4W) microbiota were assigned to 194 bacterial taxa, whereas the control group (CK_4W) contained 441 bacterial taxa (Figure 4C). At the order level, the S_4W group microbiota was dominated by Flavobacteriales (36.5%), Pseudomonadales (20%) and Staphylococcales (11%). In contrast, the CK_4W group was primarily dominated by Flavobacteriales, which accounted for more than 50% of the detected orders (Figure 4D). Significant differences were observed in the abundance of *Elizabethkingia*, *Chryseobacterium*, *Acinetobacter*, *Sediminibacterium* and *Clostridium_sensu_stricto_1* abundance between the two groups. Except for *Elizabethkingia*, the abundance of other bacterial families in the S_4W group was significantly higher than in the CK_4W group (Figure 4E). The LEfSe analysis identified distinct dominant taxa at various taxonomic levels within each group. At the genus level, the CK_4W group comprised 18 dominant genera, including *Elizabethkingia*, *Microbacterium*, and *Leucobacter*. In contrast, the S_4W group exhibited 10 dominant genera, such as *Acinetobacter*, *Chryseobacterium*, *Staphylococcus*, and *Sphingomonas* (Figure 4F). In the adult mosquito group, a total of 758,124 sequences were retained after quality filtering, resulting in the identification of 2527 OTUs across both the CK_CW and S_CW groups (Table 1). From the rank abundance curves, it was observed that the richness of the midgut microbiota in adult mosquitoes colonized by *S. aureus* decreased compared with the non-colonized control group, which is consistent with the previous comparisons between groups (Figure 5A). The subsequent PCoA analysis revealed that *S. aureus* colonization did not separate the midgut microbiota of adult mosquitoes from that of the non-colonized control group (Figure 5B). Combined with the performance observed in the larvae, these findings suggest that *S. aureus* colonization has a greater impact on the midgut microbiota during the larval stage than in adult mosquitoes. Significant differences in the proportions of bacterial classes were observed between the midguts of adult mosquitoes colonized with *S. aureus* and non-colonized controls. In both groups, Enterobacterales was the dominant order (44.6% in CK_CW and 37.9% in S_CW). In addition, there were notable differences in the abundance of Lactobacillales, Flavobacteriales, and Sphingobacteriales between the two groups (CK_CW vs. S_CW: 18.4% vs. 0.9%; 4.3% vs. 30.1%; 1.7% vs. 18.5%, respectively) (Figure 5C). At the family level, the composition of bacterial families in the midgut was significantly influenced by *S. aureus* colonization. The S_CW group exhibited a reduction in the number of bacterial families, with only 14 unique families identified (Figure 5D), indicating a decrease in microbial diversity following colonization. The proportion of Moraxellaceae was significantly higher in the midgut samples from the CK_CW group compared with those from the S_CW group (Welch’s *t*-test, *p* < 0.05, Figure 5E). At the genus level, *Corynebacterium* was absent in the S_CW group, suggesting that *S. aureus* colonization led to the loss of this genus (Welch’s *t*-test, *p* < 0.05, Figure 5F). Based on these findings, the LEfSe analysis recognized 180 taxa with significant differences between the CK_CW and S_CW groups, revealing a respective 145 and 35 distinct taxa at different taxonomic levels (Figure 5G), a significantly higher number compared with the larval group.

**Figure 6:**
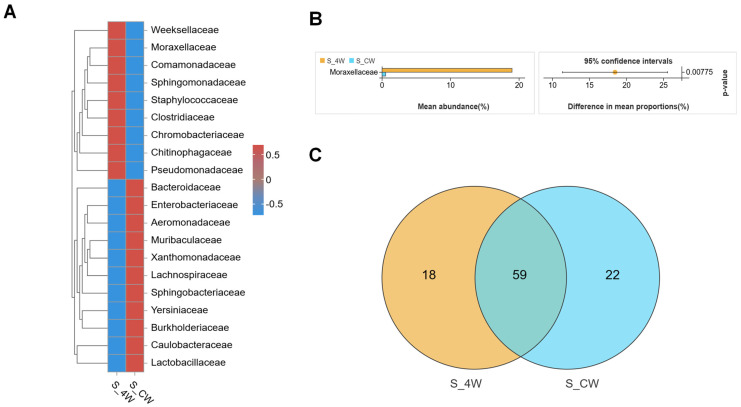
Compositional analysis of midgut bacterial communities in *Aedes aegypti* fourth-instar larvae and adults colonized with *S. aureus*: (**A**) Heatmaps of the relative abundances between the S_4W and the S_CW at the family level; (**B**) relative abundances of the bacterial families with significant differences among samples from the two groups (*p* < 0.05); (**C**) comparative analysis of Venn plots between the two groups at the family level.

Following *S. aureus* colonization, distinct differences in the midgut microbiota were observed between fourth-instar larvae and adult mosquitoes, reflecting changes associated with progression through the life stages. Taxonomic analysis at the family level, as illustrated in the species abundance heatmap, revealed increased abundances of families such as Weeksellaceae, Moraxellaceae, Staphylococcaceae, Chromobacteriaceae, and Comamonadaceae in the S_4W larvae group. In contrast, the S_CW adult group exhibited higher abundances of Enterobacteriaceae and Sphingobacteriaceae (Figure 6A). Notably, the abundance of Moraxellaceae was significantly higher in the S_4W larvae compared with the S_CW adults (S_4W vs. S_CW: 19% vs. 0.5%, Welch’s *t*-test, *p* = 0.007, Figure 6B), suggesting that members of this family may contribute to pathogen suppression through mechanisms such as competitive exclusion. A comparative analysis using a Venn diagram identified a core microbiota consisting of 59 bacterial families shared between the larval and adult stages, with each stage also containing distinct bacterial families. The S_4W group uniquely harbored 18 families, including Thermicanaceae, Veillonellaceae and Hydrogenophilaceae, while the S_CW group uniquely contained 22 families, such as Eggerthellaceae, Erysipelatoclostridiaceae, and Sutterellaceae (Figure 6C).

These findings demonstrate that *S. aureus* colonization induces significant shifts in the microbial communities of both larval and adult stages, with specific bacterial families associated with each life stage, potentially influencing the host’s immune response and susceptibility to infection.

### 3.3. Structures of Midgut Bacterial Community in Larvae and Adults Infected with B. bassiana

**Figure 7:**
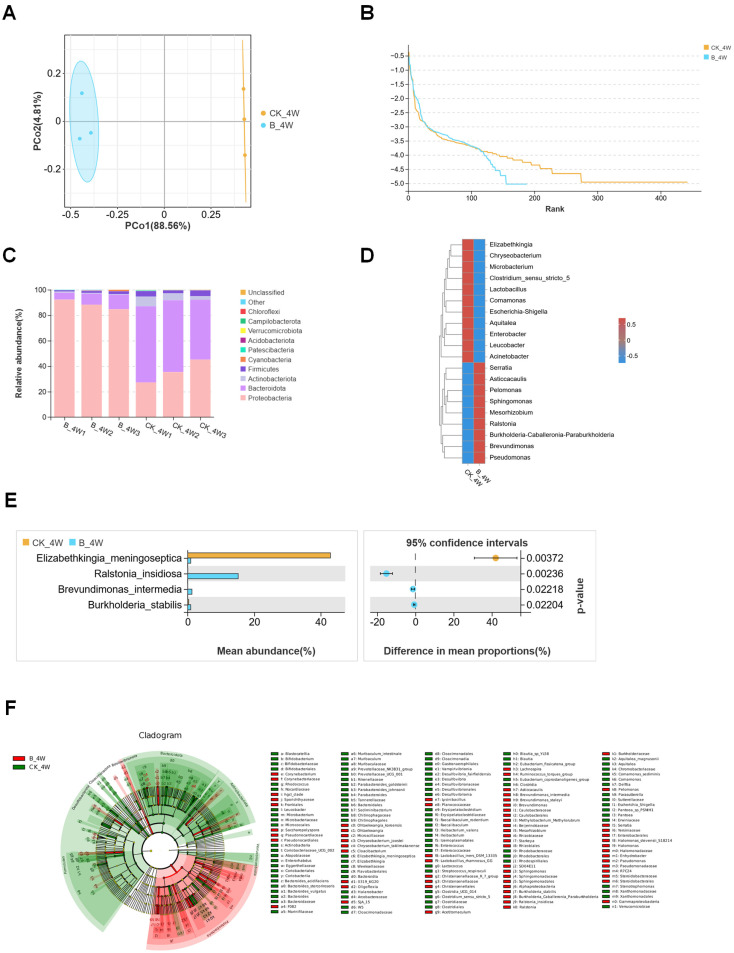
Composition and diversity of midgut bacterial microbiota in the fourth-instar larvae of *Aedes aegypti* infected with *B. bassiana*: (**A**) PCoA illustrating differences in the midgut microbiota between the CK_4W and B_4W groups; (**B**) rank abundance curve; (**C**) community analysis of midgut bacteria between the CK_4W and the B_4W, with the color-coded bar plot showing the average bacterial OTU distribution of the two groups; (**D**) heatmaps displaying the relative abundances of the top 25 genus; (**E**) relative abundances of the species with significant differences between the two groups (*p* < 0.05); (**F**) LEFse analysis of intergroup microbial differences, evolutionary branching diagram with circles radiating from the center representing classification levels from domain to species. Each small circle at different classification levels represents a species level, with the diameter of the circle proportional to its relative abundance.

A total of 673,509 reads were obtained from six samples of *Ae. aegypti* larvae, grouped into 1596 OTUs (Table 1). PCoA analysis revealed significant differences in the bacterial communities between the control CK_4W group and the infected B_4W group (Figure 7A). Additionally, the rank abundance curves indicated a reduction in bacterial community richness in the infected B_4W group (Figure 7B). In the lab-reared control group of fourth-instar larvae, the majority of the OTUs were assigned to Proteobacteria and Bacteroidota. In contrast, the OTUs from fourth-instar larvae infected with *B. bassiana* (B_4W group) exhibited reduced bacterial diversity and lower overall bacterial abundance, with *Proteobacteria* being the most abundant (Figure 7C), further indicating that *B. bassiana* infection influences the composition of the midgut microbiota.

A genus-level community heatmap illustrated the 25 most abundant genera in both groups, with *Serratia*, *Ralstonia*, *Brevundimonas*, *Burkholderia-Caballeronia-Paraburkholderia* and *Mesorhizobium* significantly more abundant in the B_4W group than in the CK_4W group (Figure 7D). At the species level, the CK_4W group had a significantly higher proportion of *Elizabethkingia_meningoseptica* (CK_4W vs. B_4W: 42.9% vs. 0.9%), but lower proportions of *Ralstonia_insidiosa* (0.03% vs. 15.2%), *Brevundimonas_intermedia* (0.001% vs. 1.263%) and *Burkholderia_stabilis* (0.29% vs. 0.90%) compared with the B_4W group larval midguts (Welch’s *t*-test, *p* < 0.05, Figure 7E). LEfSe analysis was conducted to identify the microbiota in the midgut with the most pronounced inter-group differences. The results reveal that LEfSe identified 166 taxonomic units with significant differences between the CK_4W and B_4W groups, including 31 and 24 genera, respectively (Figure 7F).

**Figure 8:**
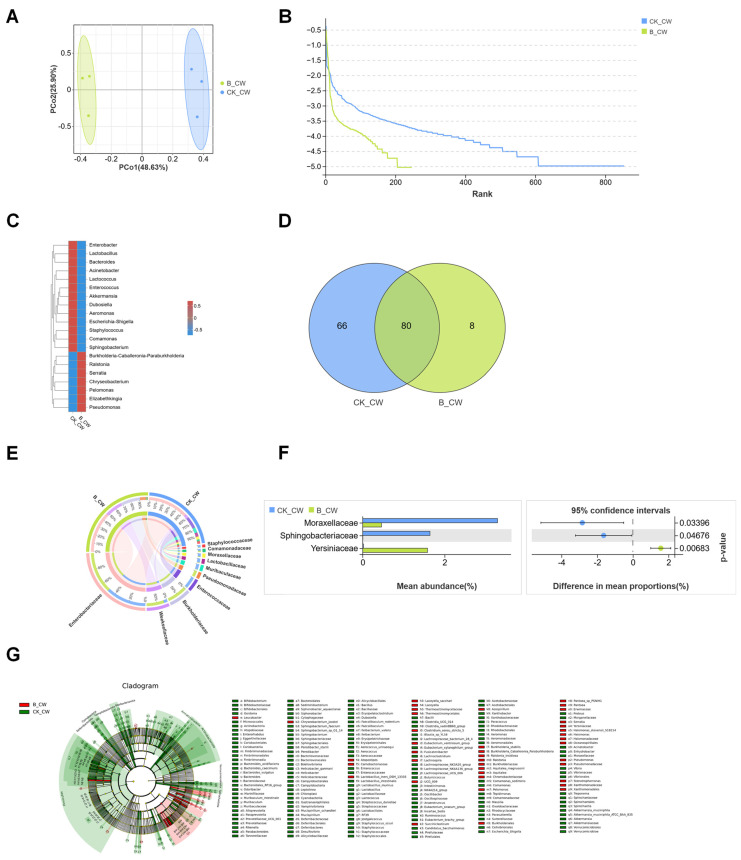
Composition and diversity of midgut bacterial microbiota in the adult mosquitoes of *Aedes aegypti* infected with *B. bassiana*: (**A**) PCoA of midgut microbiota differences between the CK_CW and B_CW groups; (**B**) rank abundance curve; (**C**) heatmaps displaying the relative abundances of the top 20 genus; (**D**) comparative analysis of Venn plots between the two groups at the family level; (**E**) community analysis of midgut bacteria between the two groups, with the color-coded Circos plot shows the average bacterial OTU distribution of the two groups; (**F**) relative abundances of the families, with significant differences between the two groups (*p* < 0.05); (**G**) LEFse analysis of intergroup microbial differences, evolutionary branching diagram with circles radiating from the center representing classification levels from domain to species. Each small circle at different classification levels represents a species level with the diameter of the circle proportional to its relative abundance.

In the adult group, after filtering the samples for quality, 757,271 reads were yielded, leading to the identification of 2747 unique OTUs (Table 1). PCoA analysis was performed to investigate the differences in bacterial community structures between the CK_CW and B_CW groups. The findings reveal a distinct aggregation of midgut composition structures between these two groups (Figure 8A), indicating that *B. bassiana* infection affects midgut microbiota composition. The subsequent rank abundance curves reveal results similar to those observed in other groups, indicating that *B*. *bassiana* infection affects the richness and evenness of the midgut microbiota (Figure 8B). Indicator species analysis revealed that, at the genus level, the abundance of *Burkholderia-Caballeronia-Paraburkholderia* (25.6%), *Elizabethkingia* (19.4%) and *Chryseobacterium* (8.9%) was higher in the B_CW group compared with the CK_CW group (Figure 8C). At the family level, Halomonadaceae, Leptotrichiaceae, Veillonellaceae, Fusobacteriaceae, Mycoplasmataceae, Pyrinomonadaceae, Carnobacteriaceae and Thermoactinomycetaceae were detected in adult mosquitoes infected with *B. bassiana* (Figure 8D). Moreover, Enterobacteriaceae was present in all mosquitoes with similar abundance in both groups (CK_CW vs. B_CW: 44.5% vs. 45.4%; Figure 8E), while Sphingobacteriaceae and Yersiniaceae were specifically associated with *B. bassiana* presence (Welch’s *t*-test, *p* = 0.00683; Figure 8F). LEfSe analysis of the CK_CW and B_CW groups revealed that the CK_CW group contained 58 significantly different genera, including *Enterococcus*, *Lactobacillus*, and *Acinetobacter*, while the B_CW group had only 19 such genera, including *Pseudomonas*, *Ralstonia*, and *Serratia*, a number significantly lower than that of the CK_CW group (Figure 8G).

**Figure 9:**
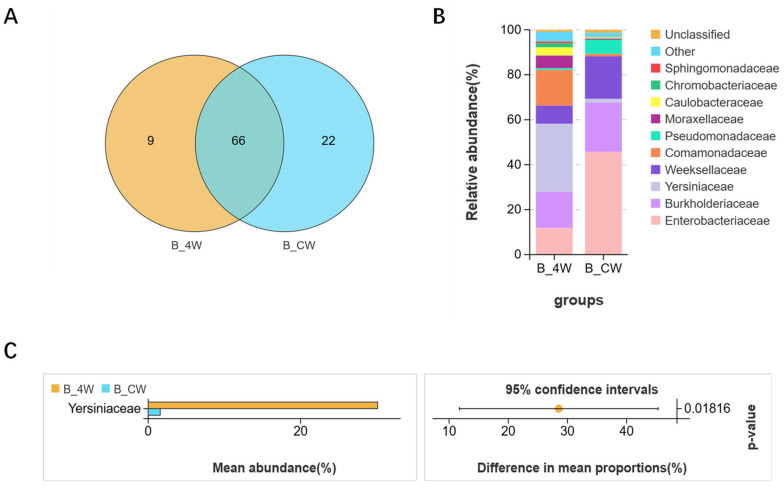
Compositional analysis of midgut bacterial communities in *Aedes aegypti* fourth-instar larvae and adults infected with *B. bassiana*: (**A**) comparative analysis of Venn plots between the B_4W and B_CW at the family level; (**B**) community analysis of midgut bacteria between the two groups, with the color-coded bar plot showing the average bacterial family distribution for both groups; (**C**) relative abundances of the bacterial families with significant differences among samples from the two groups (*p* < 0.05).

The midgut microbial shifts throughout the life cycle of mosquitoes infected with various pathogens are notably distinct. Specifically, in *Ae. aegypti* infected with *B. bassiana*, the midgut microbiota undergoes compositional changes corresponding to developmental stages, which are further influenced by the specific type of pathogen involved. A Venn diagram analysis at the family taxonomic level identified 66 bacterial families shared between the B_4W and B_CW groups. However, the B_4W group harbors significantly fewer unique families, comprising only 9 families, while the B_CW group contained 22 distinct families, such as Deinococcaceae, Porphyromonadaceae, Gemmatimonadaceae, and Hyphomicrobiaceae (Figure 9A). As *B. bassiana* infection processed, there was a marked increase in the diversity of microbial species within the midgut of adult mosquitoes compared with larvae. Additionally, new bacterial families emerged in the adult stage that were absent in the larval stage.

At the family level, a significant difference was observed in the dominant bacterial families between the B_4W and B_CW groups. In the B_4W group, the most prevalent families are Yersiniaceae (30.2%), Burkholderiaceae (16.2%), and Comamonadaceae (15.8%). In contrast, the B_CW group was dominated by a predominance of Enterobacteriaceae (45.6%), Burkholderiaceae (22%), and Weeksellaceae (19%) (Figure 9B). Heatmap analysis of species abundance and differential abundance assessments further demonstrated a notable reduction in the abundance of Yersiniaceae in adult mosquitoes following infection with *B. bassiana* (Figure 9C).

These findings suggest that *B. bassiana* infection induces distinct shifts in the midgut microbiota, with important implications for microbial diversity and host–pathogen interactions across life stages.

### 3.4. Comparative Analysis of the Composition of Fourth-Instar Larvae and Adult Mosquito of Midgut Microbiota in Ae. aegypti Mosquitoes

**Figure 10:**
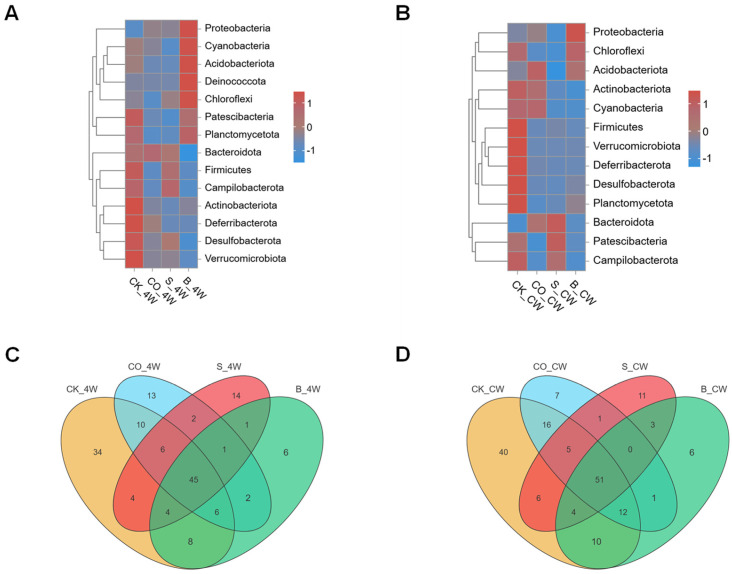
Composition analysis of midgut bacterial community in fourth-instar larvae and adult mosquito colonized with *E. coli* and *S. aureus*, and infected with *B. bassiana*: (**A**,**B**) Heatmap displaying the relative abundance of four groups at the phylum level; (**C**,**D**) comparative analysis of four groups of Venn maps at the family level.

To elucidate the impact of microorganism colonization on microbial community shifts across life stages, a comparative analysis was conducted on the midgut microbiota in fourth-instar larvae and adult *Ae. aegypti* mosquitoes. The analysis included the CK_4W, CO_4W, S_4W, and B_4W groups, revealing distinct microbial community structures at the phylum level. In the CK_4W group, the microbiota was predominantly composed of Firmicutes (19%) and Actinobacterota (3.3%). No significant differences were observed between the CO_4W and S_4W groups; however, the B_4W group, which was infected with *B. bassiana*, exhibited a marked increase in the abundance of Proteobacteria (88.6%) (Figure 10A). These results suggest a strong influence of *B. bassiana* infection on the larval microbiota composition.

Distinct microbial profiles were also observed between adult mosquitoes and fourth-instar larvae. In the adult CK_CW group, Firmicutes dominated the microbiota (26.9%), while the CO_CW and S_CW groups exhibited higher levels of Bacteroidota (40.1% and 52.1%, respectively). The B_CW group, which was infected with *B. bassiana*, showed a pronounced increase in Proteobacteria (78.5%), mirroring the changes observed in the larval group following infection (Figure 10B). These findings highlight the variability in microbial responses to pathogenic challenges across different life stages of the mosquito.

Further analysis revealed that, while many bacterial families were shared between the larval and adult mosquito groups, the control groups exhibited a greater number of unique bacterial families than the experimental groups. The CO_4W, S_4W, and B_4W groups contained 13, 14, and 6 unique families, respectively (Figure 10C). In the adult mosquito group, the CK_CW group had the highest number of unique families, with 40 species, followed by the S_CW group, including 11 unique species, such as Anaerococcus, Finegoldia, Cardiobacteriaceae, and Pasteurellaceae. Additionally, the CO_CW group contained seven unique bacterial families, while the B_CW group had the fewest endemic bacterial families, comprising only six species, Halomonadaceae, Leptotrichiaceae, Mycoplasmataceae, Pyrinomonadaceae, Haliangiaceae, and Thermoactinomycetaceae (Figure 10D). These results suggest that microorganism colonization may reduce the diversity of the midgut microbiota, disrupting the microbial equilibrium within the midgut ecosystem.

Overall, the findings indicate that *E. coli* and *S. aureus* colonization, as well as *B. bassiana* infections, induce adaptive changes in the midgut microbiota, which are closely associated with the specific type of microorganism. These microbial shifts likely reflect the host’s response to infection, with different pathogens exerting unique influences on the microbial community to fulfill essential physiological functions.

### 3.5. Functional Analysis of Mosquito Midgut Microbiota During Microorganism Colonization

**Figure 11:**
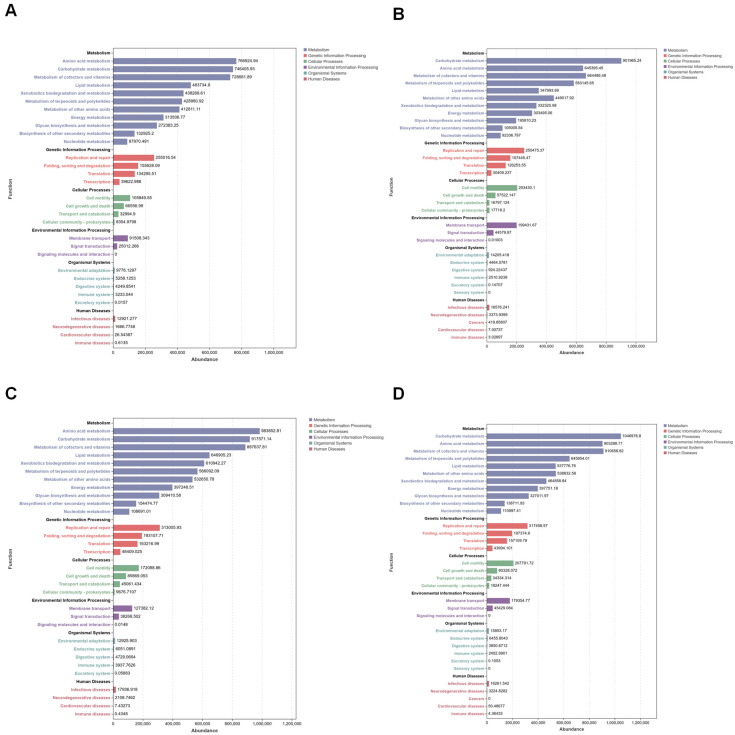
KEGG pathways at level 2 for the samples colonized with *E. coli* and for non-colonized controls. The vertical axis lists the KEGG pathways at different levels, and the length of each column indicates the corresponding functional abundance in the pathway: (**A**) Bar plots of CK_4W; (**B**) CK_CW; (**C**) CO_4W; (**D**) CO_CW.

**Figure 12:**
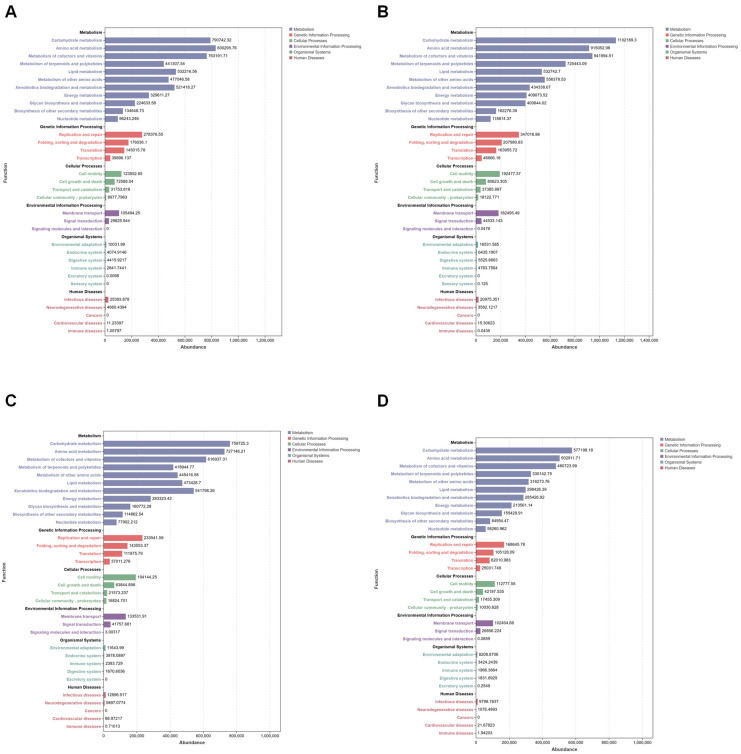
KEGG pathways at level 2 for the samples colonized with *S. aureus* and for non-colonized controls, as well as for those infected with *B. bassiana* and for non-infected mosquitoes. The vertical axis lists the KEGG pathways at different levels, and the length of each column indicates the corresponding functional abundance in the pathway: (**A**) Bar plots of S_4W; (**B**) S_CW; (**C**) B_4W and (**D**) B_CW group.

Using the 16S amplicon dataset, this study applied PICRUSt2 to predict functional pathways based on KEGG pathway metadata, with NSTI scores consistently within 0.5. A total of 33 functional pathways were identified, the majority of which were related to metabolic processes. Across all experimental groups, the most abundant pathways at KEGG Level 2 included carbohydrate metabolism, amino acid metabolism, and the metabolism of cofactors and vitamins (Figure 11 and Figure 12).

To thoroughly assess the differences in the microbiota’s functional composition between groups, Welch’s *t*-test (significance threshold set at *p* < 0.05) was performed to compare the relative abundance of KEGG level B pathways. The results revealed enrichment in 11 metabolic pathways, with a particular emphasis on carbohydrate metabolism, amino acid metabolism, and the metabolism of cofactors and vitamins.

**Figure 13:**
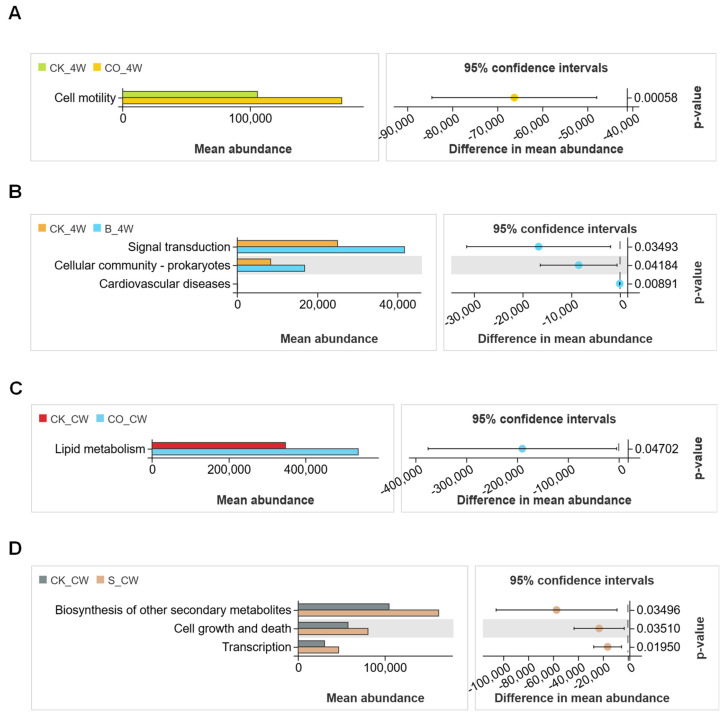
KEGG pathways at level 2 for the samples colonized with *E. coli* and *S. aureus,* and non-colonized controls, as well as for samples infected with *B. bassiana* and for non-infected mosquitoes: (**A**) Extending error bar plot indicating differences in functional profiles between the CK_4W and CO_4W groups; (**B**) CK_4W and B_4W groups; (**C**) CK_CW and CO_CW groups; and (**D**) CK_CW and S_CW groups.

In the larval groups, *E. coli* colonization in the CO_4W group led to a significant increase in the cell motility pathway compared with the CK_4W group (Welch’s *t*-test, *p* < 0.05; Figure 13A). Infection with *B. bassiana* in the B_4W group resulted in elevated pathways related to signal transduction, cellular community prokaryotes, and cardiovascular diseases (Welch’s *t*-test, *p* < 0.05; Figure 13B).

In adult mosquito groups, when compared with the CK_CW group, the CO_CW group exhibited a marked increase in relative abundance in lipid metabolism after colonization with *E. coli* (Welch’s *t*-test, *p* < 0.05; Figure 13C). In contrast, the S_CW group showed significant upregulation in pathways related to the biosynthesis of secondary metabolites, cell growth and death, and transcription following *S. aureus* colonization (Welch’s *t*-test, *p* < 0.05; Figure 13D). The CK_CW group and the B_CW group did not show significant differences in their functional profiles (Welch’s *t*-test, *p* > 0.05).

These results suggest that, while there are notable differences in functional pathways in response to different colonization, the overall differences in predicted functions between the larval and adult groups were relatively smaller under the same colonization conditions. This indicates that, while colonization type strongly influences the functional potential of the microbiota, the life stage of the mosquito has a comparatively minor effect on functional shifts within the microbiota. Using PICRUSt2, we predicted functional pathways based on 16S rDNA sequencing data. These predictions provide insights into potential metabolic activities but require experimental validation.

## 4. Discussion

This study aimed to investigate the alterations in the midgut microbiota of *Ae. aegypti* mosquitoes following colonization with *E. coli* and *S. aureus*, as well as infection with *B. bassiana*. Additionally, the role of the midgut microbiota was explored in mosquito innate immunity. Our findings provide a better understanding of the roles of different microorganisms. These roles could potentially lead to a decrease in microbiota diversity in the host. The composition of the mosquito midgut microbiota is influenced by several key factors during the larval stage, including environmental microorganisms, feeding behavior, biological agents, and pathogen infections [39,40]. In this study, the observations revealed that Flavobacteriales, Burkholderiales, Enterobacterales, Pseudomonadaceae, Sphingobacteriales and Lactobacillales are the largest contributors to the midgut microbiota in both colonized and non-colonized mosquitoes. This finding is consistent with previous studies on mosquito microbiota [41,42], highlighting the stability of these bacterial groups across different conditions.

However, our study demonstrates that microorganism colonization appears to significantly alter the microbiota composition. For instance, a comparison between the CK_4W and CO_4W groups revealed that Comamonadaceae was more prevalent in the colonized CO_4W, while it was present in smaller quantities in the control CK_4W group (Figure 1E). At the species level, the abundance of *Escherichia shigella* also showed significant differences between the two groups (Figure 1E), underscoring the direct impact of *E. coli* colonization on the midgut microbiota. Similar patterns have been reported in other studies, where Comamonadaceae was identified as a key microbiota component in both *Microsporidian*-infected and non-infected mosquitoes [40].

Upon *E. coli* colonization, there is an observed increase in the abundance of Enterobacteriaceae, particularly *Escherichia shigella* species, which are closely related to *E. coli*. This shift likely disrupts the native microbial balance, potentially increasing the mosquito’s susceptibility to other pathogens [21,43]. Specifically, the presence of *E. coli* fosters an increase in certain bacterial genera, including opportunistic pathogens like *Serratia*, which are typically not dominant in a healthy gut microbiota. This increase may compromise the mosquito’s defense mechanisms [21]. These changes suggest that certain microorganisms undergo adaptive shifts in response to *E. coli* colonization, potentially impacting mosquito physiology and pathogen susceptibility.

The impact of colonization on microbiota composition is further highlighted in the CK_4W group, where *Elizabethkingia* was found in large proportions but significantly decreased in abundance in the S_4W and B_4W groups (Figure 4E and Figure 7E). *Elizabethkingia*, a genus of gram-negative bacilli found globally [44], is frequently described as a dominant gut endosymbiont of *anophelines* [45,46], and studies suggest that the mosquitoes are the reservoirs of *Elizabethkingia* [47]. In adult mosquitoes, the microbiota composition between the CK_CW and CO_CW groups remained highly similar (Figure 2C). However, adult mosquitoes colonized with *S. aureus* exhibited a decrease in Moraxellaceae and an absence of *Corynebacterium* compared with the control group (Figure 5E,F). Various studies indicate that Moraxellaceae predominates in the midgut microbiota of adult mosquitoes, especially increasing during or after blood feeding [48,49]. *Corynebacterium*, known as a top ten human skin commensal, is not only a mosquito attractant but also a major component of the midgut microbiota in *Anopheles stephensi*, with its abundance increasing after *cypermethrin* treatment [50,51]. The absence of *Corynebacterium* after *S. aureus* colonization warrants further investigation into whether changes in its abundance affect mosquito feeding behavior and pathogen transmission. Beyond compositional changes, *S. aureus* colonization leads to notable reductions in the overall diversity of the mosquitos’ midgut microbiota, including a decrease in overall diversity and reduction of beneficial bacterial populations such as Lactobacillaceae and Acetobacteraceae. The colonization also causes an increase in Enterobacteriaceae, potentially due to immune responses that favor pathogenic bacteria. Additionally, *S. aureus* colonization increases gut permeability, [52] These findings highlight the intricate relationship between microorganism colonization and gut microbiota composition in mosquitoes.

Similarly, *B. bassiana* infection induces significant shifts in the midgut microbiota composition. At the family level, *B. bassiana* infection in the B_CW group was found to be associated with a decrease in Sphingobacteriaceae and an increase in Yersiniaceae (Figure 8F). Members of the Yersiniaceae family typically engage in complex interactions with various organisms and environments.

Additionally, as the duration of pathogen infection continues, the abundance of Yersiniaceae in mosquitoes gradually decreases, possibly due to their depletion in the process of pathogen suppression. Previous studies have indicated that *Yersinia* species can induce pyroptosis, a form of programmed cell death that facilitates the recruitment of immune cells and the clearance of invading pathogens through the release of pro-inflammatory factors [53]. Sphingobacteriaceae is known to combat fungal invasion and exhibit fungicidal activities or induce antagonistic traits in other bacterial taxa [54]. This suggests that Sphingobacteria play a role in resisting fungal damage in mosquitoes and are continuously utilized to combat fungal harm to the insect’s body. Additionally, Yersiniaceae, identified in fungus-growing termites, also respond to fungal invasion, aligning with our findings [55].

Overall, infection with *B. bassiana* significantly alters the midgut microbiota of mosquitoes, leading to adaptive changes in response to fungal invasion. The presence of *B. bassiana* induces shifts in the relative abundance of various microbial taxa, with increases in bacterial genera such as *Providencia* and *Enterococcus*, which are known for their antifungal properties. Previous studies have demonstrated that the bacteria can inhibit the germination and growth of *B. bassiana*, thereby stabilizing the midgut microbiota and providing protection against the fungal pathogen [56]. These adaptive changes in the midgut microbiota under the influence of *B. bassiana* may indicate complex interactions between fungal pathogens and the mosquito midgut microbiota, potentially affecting mosquito health and pathogen resistance.

Analysis of the treatment groups for fourth-instar larvae and adult mosquitoes revealed that both life stages experience a reduction in gut microbiota richness following pathogen infection. Upon colonization and infection, the immune system of mosquitoes is activated, resulting in the production of immune factors such as antimicrobial peptides. While these immune responses primarily target pathogens, they can also inhibit the symbiotic bacteria within the gut, leading to dysbiosis—a microbial imbalance [15]. Furthermore, pathogen infection may compromise the integrity of intestinal barriers in mosquitoes, increasing susceptibility to environmental and endogenous stressors. This alteration is associated with a decline in specific bacterial populations and an overall reduction in microbial diversity [14].

Pathogen infection also intensifies competition for nutritional resources within the mosquito gut, particularly in its nutrient-limited environment. As pathogens proliferate, they can dominate this competition, leading to a decrease in the abundance of beneficial symbiotic microorganisms [57]. Additionally, pathogen infection can alter the metabolic processes of mosquitoes, influencing the physicochemical conditions of the gut environment, including pH and oxygen concentration, thereby further exacerbating dysbiosis. For instance, certain bacterial species may struggle to survive under infectious conditions, leading to a further reduction in the overall diversity of the microbial community [58].

While both larval and adult stages of *Ae. aegypti* exhibited significant microbiota alterations upon microorganism colonization, the nature and extent of these changes varied. For instance, *E. coli* colonization led to a substantial increase in Comamonadaceae in larvae (CO_4W) but had a more modest impact on adults (CO_CW), suggesting stage-specific microbial responses. Conversely, *B. bassiana* infection resulted in a marked increase in Yersiniaceae in adults (B_CW) compared with larvae (B_4W), indicating differential adaptive mechanisms across life stages. The differential responses observed between larval and adult stages emphasize the complexity of host–microbiota–pathogen interactions. In larvae, the rapid development and environmental exposures may necessitate more pronounced microbial shifts to accommodate pathogen presence, whereas adult mosquitoes, with more established microbiota, exhibit subtler changes that reflect mature immune and physiological states.

The predictions of microbiome functional content, derived from 16S rDNA gene sequencing, indicate that infections can induce shifts in the metabolic activity of the mosquito microbiome. Specifically, pathways associated with carbohydrate metabolism, amino acid metabolism, and the metabolism of cofactors and vitamins appear more abundant in infected mosquitoes. Additionally, the metabolism of terpenoids and polyketides, lipid metabolism and other amino acids pathways are comparatively enriched in the microbiota of infected hosts. These changes suggest that infections alter the metabolic landscape of the mosquito microbiome, which could, in turn, influence mosquito physiology and immune responses.

Previous research has also shown that carbohydrate metabolism is essential for the moth *Bombyx mori* and the fly *Sarcophaga crassipalpis* to wake from diapause [59,60,61,62]. Enzymes involved in carbohydrate and lipid metabolism facilitate reactions that break down food, release stored energy, and synthesize the main energy storage of living organisms [63,64]. Therefore, the effects of various metabolic pathways observed in our study’s mosquitoes may explain the enrichment of these pathways in the KEGG functional analysis. For example, *E. coli* colonization was found to be associated with increased cell motility and lipid metabolism pathways, particularly bacterial chemotaxis and flagellar assembly, indicating a shift in metabolic homeostasis (Figure 13A,C). These findings support the idea that infected pathogens can disrupt the metabolic homeostasis of organisms [65]. Furthermore, our research revealed a relative enrichment in the biosynthesis of secondary metabolites, along with pathways involved in cell growth, death, and transcription, within the microbiota of *S. aureus*-colonized mosquitoes (Figure 13D). *B. bassiana* infection led to increases in signal transduction and cellular community pathways (Figure 13B), reflecting significant metabolic adjustments in response to fungal infection. These pathogen-specific shifts in metabolic function illustrate the adaptive responses of the mosquito microbiome to diverse infections, potentially affecting mosquito health, immune resilience, and vector competence. However, these functional predictions are based on computational models and should be validated through metagenomic or transcriptomic analyses in future studies.

These findings demonstrate the intricate relationship between pathogen infection, microbial colonization, and mosquito midgut microbiota, highlighting how different microorganisms induce specific alterations in microbiota composition and function. This dynamic interplay suggests that each pathogen can uniquely influence the microbial community within the mosquito midgut, potentially altering host immunity and overall physiology. Growing evidence supports the existence of a critical metabolic interdependence between the microbiome and the mosquito vector that may have detrimental effects if this metabolic relationship is perturbed [65].

Our research indicates that the mosquito microbiota adapts to infection by specific pathogens through alterations in metabolic pathways, which may contribute to maintaining or reestablishing metabolic homeostasis. These pathogen-specific adaptations in the microbiota provide insights into how mosquitoes respond to microbial challenges and may inform targeted interventions in vector control.

In conclusion, this study demonstrates that different microorganisms‘ colonization lead to specific changes in the mosquito midgut microbiota. A deeper understanding of these interactions is essential for developing effective, microbiome-targeted strategies to control mosquito-borne diseases and disrupt disease transmission.

## 5. Conclusions

This study demonstrates that colonization with *E. coli* and *S. aureus*, as well as infection with *B. bassiana* significantly influence the microbial community structure and activity in the midgut of *Ae. aegypti* mosquitoes. Notably, significant shifts were observed in the presence of specific bacterial taxa including Comamonadaceae, Moraxellaceae, Sphingobacteriaceae, and Yersiniaceae at the family level, as well as *Escherichia shigella*, *Elizabethkingia*, and *Corynebacterium* at the genus level.

Our research provides fundamental insights into the complex interactions between mosquito hosts, their symbiotic microbiota, common bacteria, and insect pathogenic fungi. These findings contribute to the understanding of how different pathogens influence the midgut microbiota and may inform the development of improved biological control strategies targeting mosquito vectors.

## Figures and Tables

**Table 1:** Sequencing results.

Sample Name	Raw Reads	Clean Reads	Raw Tags	Clean Tags	Chimera	Effective Tags
CK_4W1	125,826	125,725	124,117	123,665	7212	116,453
CK_4W2	124,908	124,815	123,285	122,951	9406	113,545
CK_4W3	68,129	68,079	67,193	66,989	5427	61,562
CK_CW1	135,349	134,439	119,449	118,428	5363	113,065
CK_CW2	128,135	127,512	117,013	116,482	5549	110,933
CK_CW3	131,175	130,061	116,129	114,688	6126	108,562
CO_4W1	123,780	123,682	122,163	121,860	5414	116,446
CO_4W2	115,998	115,899	114,404	114,090	4035	110,055
CO_4W3	128,115	128,017	126,494	126,156	6316	119,840
CO_CW1	124,746	124,120	114,681	113,974	2609	111,365
CO_CW2	121,009	120,447	112,761	111,903	4137	107,766
CO_CW3	125,995	125,335	114,293	113,793	9011	104,782
S_4W1	111,938	111,842	110,280	109,916	2352	107,564
S_4W2	121,867	121,752	120,144	119,668	2995	116,673
S_4W3	113,832	113,733	112,242	111,945	2697	109,248
S_CW1	116,513	116,406	115,066	114,643	2828	111,815
S_CW2	132,052	131,957	130,225	129,834	6147	123,687
S_CW3	117,870	117,749	116,260	115,856	5566	110,290
B_4W1	117,559	117,515	116,836	116,708	5001	111,707
B_4W2	113,668	113,621	113,096	112,751	4267	108,484
B_4W3	123,419	123,373	122,707	121,556	4283	117,273
B_CW1	113,555	113,515	113,066	112,955	6063	106,892
B_CW2	130,802	130,749	130,044	129,882	9759	120,123
B_CW3	121,045	120,995	120,423	120,266	7759	112,507

## Data Availability

The original data presented in the study are openly available in NCBI under accession number PRJNA1188320.
